# A Case of Multiple Myeloma Misdiagnosed as Seronegative Rheumatoid Arthritis and Review of Relevant Literature

**DOI:** 10.1155/2018/9746241

**Published:** 2018-10-11

**Authors:** Scott Schoninger, Yamen Homsi, Alexandra Kreps, Natasa Milojkvovic

**Affiliations:** ^1^College of Medicine, SUNY Downstate Medical Center, 450 Clarkson Avenue, Brooklyn, NY 11203, USA; ^2^Division of Rheumatology, SUNY Downstate Medical Center, 450 Clarkson Avenue Box 42, Brooklyn, NY 11203, USA; ^3^Division of Hematology, University of Arkansas for Medical Sciences, 4301 West Markham Street, Little Rock, Arkansas 72205, USA

## Abstract

Multiple myeloma (MM) is a malignant plasma cell proliferation producing large numbers of monoclonal immunoglobulins. Typical MM symptoms include anemia, renal failure, hypercalcemia, and bone pain. Atypical symptoms have rarely been reported in the literature. We report a case of a 58-year-old male who presented with symmetrical inflammatory polyarthritis and was misdiagnosed with seronegative rheumatoid arthritis (RA). After failing many RA treatments and with further workup, the diagnosis of MM was made. This rare manifestation of MM carries a diagnostic challenge and causes a significant delay in treating such patients. Here, we report this unusual initial presentation with review of several cases in the English literature describing similar presentations.

## 1. Introduction

Multiple myeloma (MM) is a clonal plasma cell malignancy that accounts for approximately 10% of hematologic malignancies [1]. In the United States, the lifetime risk of getting MM is 1 in 132 (0.76%) [2]. In patients presenting at under 60 years of age, the 10-year survival rate is about 30% [3]. The diagnosis of MM is often delayed due to lack of recognition of the most common presenting symptoms, such as fatigue, anemia, renal insufficiency, and hypercalcemia. An atypical manifestation could put an even greater challenge on the diagnosis and subsequently cause a delay in treatment. We describe a 58-year-old male who presented with symmetrical inflammatory synovitis as a first sign of MM, and we report the relevant literature.

## 2. Case Presentation

A 58-year-old male was referred to the rheumatology clinic for evaluation of arthralgia of the hands, wrists, and elbows. The patient's symptoms started six months prior and gradually worsened. The patient endorsed swelling of the hands and wrists, difficulty making fists, as well as morning stiffness lasting more than thirty minutes. He denied any constitutional symptoms such as fevers, chills, weight loss, decreased appetite, or night sweats. Review of systems was negative for alopecia, dry eyes, dry mouth, mouth sores, and skin rash. The patient also denied any recent travel, tick bites, or sick contacts. He never smoked and consumed alcohol on an occasional basis. The patient had a past medical history of osteoarthritis, with a surgical history significant for multiple procedures, including bilateral shoulder replacement for severe osteoarthritic changes, carpal tunnel repair of the right side, and laminectomy of the cervical and lumbar spine. Clinical exam revealed normal vitals, with benign head, eye, ear, nose, throat, cardiopulmonary, and abdominal exams. No lymphadenopathy or bruises were observed. Musculoskeletal exam revealed synovitis of the second through fifth metacarpophalangeal (MCP) and proximal interphalangeal regions bilaterally, with swelling and tenderness of the wrists with warmth to touch. In addition, there were 30-degree fixed contractions of the elbows. As per the patient, there was no history of psoriasis or nail changes, which was confirmed on physical exam as well. Laboratory data showed white blood cells of 12,000/mm, hemoglobin of 9.7 g/dl, hematocrit of 30.9%, C-reactive protein of 40 mg per liter (reference value <8), and erythrocyte sedimentation rate of 50 mm per hour (reference range 0 to 15). Laboratory testing of liver function, calcium, thyroid function, uric acid, renal function, and urinalysis was normal. Other normal or negative tests included antinuclear antibody, rheumatoid factor (RF), anti-cyclic citrullinated peptide (anti-CCP) antibodies, hepatitis B panel, hepatitis C antibody, QuantiFERON-TB Gold test, and angiotensin-converting enzyme. The patient was seen by an outside rheumatologist and treated initially with prednisone 20 mg/d and methotrexate, which was quickly escalated to a dose of 20 mg/weekly, with no response after three months of treatment. He was subsequently started on antitumor necrosis factor inhibitors.

**Figure 1:**
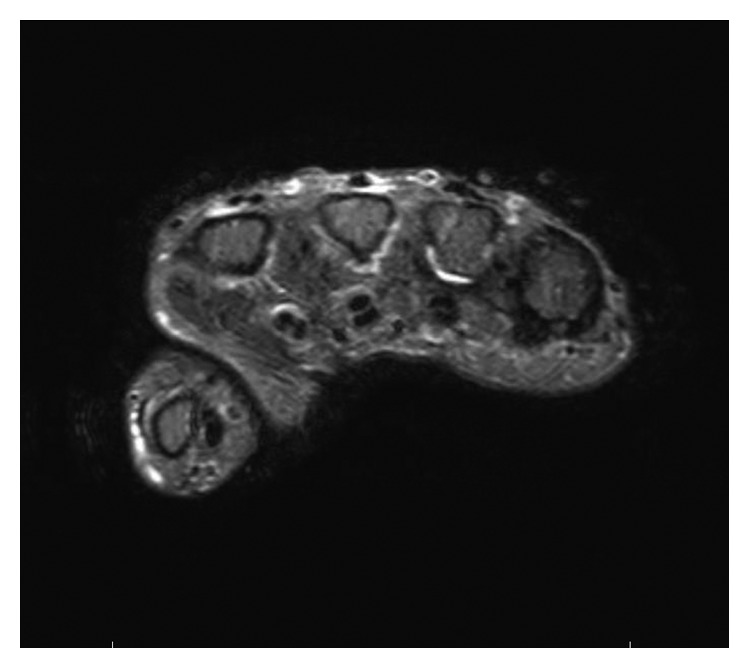
MRI of the right hand without contrast. Short T inversion recovery (STIR) showing tenosynovitis of the flexor tendons of the second and third digits and synovitis of the second, third, and fourth MCP joints.

Six months later, a follow-up visit showed persistent symptoms. Alternative anti-TNF inhibitors were tried with no improvement. Further workup included X-rays of the hands and wrists, which showed mild degenerative changes only. Chest X-ray revealed no pathology. The patient underwent MRI of the right hand, which showed synovial thickening of the radiocarpal joint and MCP joints with flexor tendon tenosynovitis, compatible with inflammatory arthropathy (Figure 1). Further questioning of the patient revealed a family history of primary amyloidosis in his mother. Serum calcium, uric acid, and creatinine remained normal during the time of RA treatment. As a result, serum protein electrophoresis (SPEP) and urine protein electrophoresis were sent. Interestingly, SPEP was positive for M-band. The patient was then referred to hematology for further evaluation and underwent a bone marrow biopsy, which was positive for more than 40% plasma cells, as well as being Congo red stain negative, findings consistent with MM. FISH analysis was positive for monosomy 13 in 88% of the cells. The patient then started treatment for MM with bortezomib and dexamethasone. Six-month follow-up showed complete resolution of joint swelling, with significant improvement in pain of the hands, wrists, and elbows. His MM remained quiescent with chemotherapy, and the patient did not require bone marrow transplant.

## 3. Discussion and Literature Review

**Figure 2:**
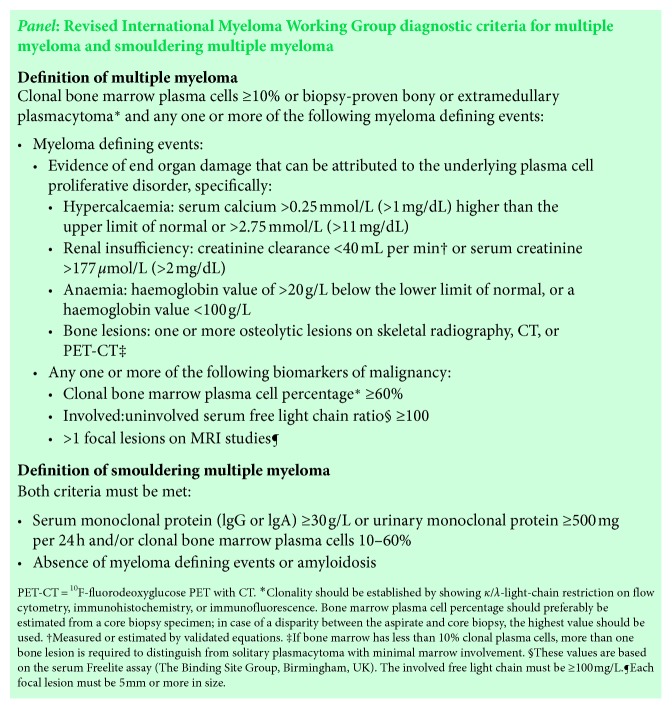
2014 Updated Diagnostic Criteria of Multiple Myeloma by the International Myeloma Working Group.

Multiple myeloma is a cytogenetically heterogeneous, clonal plasma cell proliferative disorder, which can produce a monoclonal immunoglobulin, and which accounts for approximately 1% of neoplastic diseases and 10% of hematologic cancers [1]. The American Cancer Society estimates that 30,770 new cases of MM will occur in the US in 2018, with approximately 12,770 deaths expected to occur [2]. In 2014, the International Myeloma Working Group released updated diagnostic criteria [4] (Figure 2). The incidences of presenting symptoms of MM are bone pain (58%), fatigue (32%), pathologic fracture (26 to 34%), weight loss (24%), paresthesias (5%), and fever (0.7%) [5].

Rare presenting symptoms of MM can cause a significant delay in treatment and lead to unfavorable outcomes. Our case's unusual initial presentation of MM prompted a literature review to look for other reported cases presenting as inflammatory arthritis. We have performed a systematic search of English literature on PubMed from 1991 to 2018 using the keywords “multiple myeloma” and “monoclonal gammopathy” with “peripheral inflammatory arthritis,” “rheumatoid arthritis,” and “rheumatologic diseases.” 344 articles were obtained and reviewed. The articles collected were only cases which resembled or had close presentation to our patient and are summarized in Table 1.

The largest series, reported by Jorgensen et al. in 1996, included nine patients with monoclonal gammopathy (MG), either MM or monoclonal gammopathy of uncertain significance, who developed inflammatory chronic arthritis simultaneously or after the diagnosis of MG. Interestingly, all of the patients were seronegative for rheumatoid factor, and the majority had hand and wrist involvement, including two patients who had distal interphalangeal joint involvement not typical of RA [10]. Vitali et al. published a similar series of four cases in 1991, in which, like Jorgensen et al., the MG was found prior to or during the development of arthritis. Two patients had rheumatoid-like, symmetric polyarthritis of the MCP joints and wrists [12]. DIP involvement was reported in this series as well. In addition, RF was negative in four cases.

In 2003, Fujishima et al. described one case of symmetrical polyarthralgias and multiple joint swellings with negative RF, mimicking RA. Bone marrow biopsy was consistent with MM, and synovial biopsy showed amyloid arthropathy [14]. Another similar observation of a case of MM presenting as acute interstitial nephritis and RA-like polyarthritis was reported by Ardalan and Shoja in 2007. In this case, the patient initially presented with rapidly progressive renal failure and underwent renal biopsy, which was consistent with acute interstitial nephritis [9]. Two months later, he returned with symmetric polyarthralgias of the hands and feet, with associated knee effusions. Further workup with bone marrow aspiration and biopsy revealed MM, and the RF was negative. Another interesting unusual presentation described by Molloy et al. in 2007 highlighted a case of erosive seronegative inflammatory arthritis in association with bilateral carpal tunnel syndrome as rare symptoms of MM [8]. In 2009, Alpay reported two patients with symmetric polyarthralgias of the hands, wrists, shoulders, and temporomandibular joints, both diagnosed with MM and found to have amyloid deposition seen on synovial biopsy [7].

More recently, Srinivasulu et al. reported a case series of 6 patients in 2012, 5 of which were seronegative for RF and anti-CCP antibodies and 1 was positive for both [6]. Two separate cases of MM presenting as inflammatory polyarthritis were reported by Fedric and Agarwal. Fedric described a 72-year-old woman who presented with bilateral symmetric synovitis of the knees, ankles, wrists, and metacarpophalangeal joints, who was diagnosed with MM associated with amyloidosis. Agarwal described MM in a case of a young male who presented with polyarthralgias and rash [11, 13]. A case of MM masquerading as seropositive RA with cutaneous amyloid nodules thought to be RA nodules was described by Edavalath in 2017 [15]. Most recently, Bornstein et al. published a series of four cases of various hematological malignancies mimicking rheumatic syndromes. One of these cases was a patient with seronegative inflammatory polyarthritis of the wrists, MCP and PIP joints, who was later found to have MM with restrictive cardiomyopathy, secondary to amyloidosis, and died shortly thereafter [16]. The association between monoclonal gammopathy (MG) and rheumatic disease has been investigated and showed increased prevalence of MG among patients with rheumatic diseases [17].

From our in-depth review, we have observed that the hands and wrists are the most commonly involved joints in patients with MG mimicking RA, and that almost all patients have negative RF. Of note, anti-CCP antibody was not mentioned in the older references since it was not discovered at that time. Our case illustrates an atypical presentation of MM mimicking seronegative rheumatoid arthritis, which carried a diagnostic challenge for physicians and caused a delay in the treatment of a life-threatening malignancy. Lack of response to RA treatment should prompt the physician to reexamine the initial diagnosis and think of alternatives. Increased awareness of atypical symptoms for serious diseases such as MM is critically important among health care providers and should become more emphasized in future practice.

## Figures and Tables

**Table 1:** Literature review table.

Number of patients	Affected joints	Serology	Monoclonal gammopathy diagnosis	Reference
6	Wrists and shoulders are the most commonly involved joints	Five patients were negative for RF and anti-CCP antibodies One patient was positive for RF and anti-CCP antibodies	Four patients diagnosed with MGUS and two patients diagnosed with MM	Srinivasulu [6]

2	Wrists, hands, TMJ, and shoulders	Negative RF Negative anti-CCP	MM associated with amyloid arthropathy	Alpay [7]

1	Hands, wrists, shoulders, and knees	Negative RF Anti-CCP antibodies were not reported	MM	Molloy [8]

1	Hands, knees, and feet	Negative RF Anti-CCP antibodies were not reported	MM	Ardalan [9]

9	Hands, wrists, shoulders, and knees. One case had sacroiliitis	Negative RF Anti-CCP antibodies were not reported	Two patients diagnosed with MM. Seven patients diagnosed with MGUS	Jorgensen [10]

1	DIP joints, PIP joints, wrists, knees, and ankles	Negative RF Negative anti-CCP antibodies	MM with amyloidosis	Roca [11]

4	DIP joints, PIP joints, MCP joints, and wrists	Negative RF Anti-CCP antibodies were not reported	MGUS	Vitali [12]

1	Knees	Negative RF Negative anti-CCP antibodies	MM	Agarwal [13]

1	Hands and wrists	Negative RF Anti-CCP antibodies were not reported	MM with amyloidosis	Fujishima [14]

1	Hands and wrists	Positive anti-CCP antibodies Negative RF	MM	Edavalath [15]

4	3 of the 4 patients had polyarthritis of hands and wrists	RF and anti-CCP antibodies were not reported	MM and amyloidosis	Bornstein [16]

RF: rheumatoid arthritis; anti-CCP antibodies: anti-cyclic citrullinated peptide antibodies.
